# Nurses' preparedness to care for women exposed to Intimate Partner Violence: a quantitative study in primary health care

**DOI:** 10.1186/1472-6955-11-1

**Published:** 2012-01-10

**Authors:** Eva M Sundborg, Nouha Saleh-Stattin, Per Wändell, Lena Törnkvist

**Affiliations:** 1 Center for Family and Community Medicine, Department of Neurobiology, Care Sciences and Society, Karolinska Institutet, Alfred Nobels allé 12, 145 60 Huddinge, Sweden

## Abstract

**Background:**

Intimate partner violence (IPV) has a deep impact on women's health. Nurses working in primary health care need to be prepared to identify victims and offer appropriate interventions, since IPV is often seen in primary health care. The aim of the study was to assess nurses' preparedness to identify and provide nursing care to women exposed to IPV who attend primary health care.

**Method:**

Data was collected using a questionnaire to nurses at the primary health care centres. The response rate was 69.3%. Logistic regression analysis was used to test relationships among variables.

**Results:**

Shortcomings were found regarding preparedness among nurses. They lacked organisational support e.g. guidelines, collaboration with others and knowledge regarding the extensiveness of IPV. Only half of them always asked women about violence and mostly when a woman was physically injured. They felt difficulties to know how to ask and if they identified violence they mostly offered the women a doctor's appointment. Feeling prepared was connected to obtaining knowledge by themselves and also to identifying women exposed to IPV.

**Conclusion:**

The majority of the nurses were found to be quiet unprepared to provide nursing care to women exposed to IPV. Consequences might be treatment of symptoms but unidentified abuse and more and unnecessary suffering for these women. Improvements are needed on both at the level of the organisation and individual.

## Background

### Intimate partner violence - a public health problem

Intimate partner violence (IPV) is a recognised public health problem with tremendous impact on a woman's health, both during the abuse is taking place and long after it has ceased [1-4]. According to Cutliffe and McKenna (2005), in a relationship where IPV occurs, abuse constitutes; *'physical, psychological or sexual mistreatment and/or other controlling behaviours such as economic or spiritual deprivation that are intended by the abuser to cause harm or are perceived by the victim to cause harm. It is a purposeful behaviour designed to achieve domination and control in the relationship' *[[5], p. 28]. IPV occurs in all countries irrespective of socioeconomic status, religion or culture [6,7].

When encountering women exposed to IPV, nurses should be well prepared to provide them with nursing care of high quality. Despite that, studies have shown that nurses in primary health care (PHC), are more ill-prepared to detect IPV and intervene than professionals in other areas, such as emergency and gynaecological care [8,9]. In light of such findings, special attention should be paid on improving preparedness of nurses in PHC.

To identify women exposed to IPV, it is important for health care professionals to know that IPV is a growing and multifaceted problem. Health problems caused by IPV can manifest as physical illness, sexual/reproductive dysfunction and mental disorders [7,10-14]. Also, living in a violent relationship often affects a woman's ability to trust other people [9].

### Preparedness to provide nursing care to women exposed to IPV

Improving preparedness among healthcare professionals in dealing with women exposed to IPV, appropriate attitudes and a supportive working environment are necessary [15-18]. To achieve these, proper training, continuous support, and sufficient time spent with women exposed to IPV must be in place [7,19]. Since 1980s there have been major legal reforms in Sweden. The law provides IPV victims with orders of protection and enhances the possibilities for police interventions. In addition, children living where IPV occurs are exposed to psychological abuse. In such cases, all health care personnel are obligated to involve social services but not the police. The Swedish welfare system offers women exposed to IPV a place in designated shelters that provide temporary refuge. Shelters may also break the isolation that women exposed to IPV often experience. Shelter workers are usually volunteers with experience and a developed expertise in assisting and advising women exposed to IPV.

Despite adjustments in the legal and social welfare systems to accommodate the needs of IPV victims, recent study findings have indicated that only a small percentage of women exposed to IPV are identified by healthcare staff [17]. Health problems associated with IPV are often treated as problems in their own right rather than as the result of IPV [20,21]. Consequently, when the abuse remains undetected, the women may experience a secondary victimisation and the suffering of care [22,23]. Results from studies have shown that healthcare staff often lack relevant knowledge, are uncertain about how to ask, are concerned about breaching the woman's integrity, or are unsure about which intervention should be implemented once IPV is confirmed [18,24-27]. Having said that, adequate supervision of nurses in PHC and appropriate policies nationwide could prove effective in the identification of women exposed to IPV when encountering them. However, in Sweden, proper supervision of nurses in PHC is not usually available nor is there any policies specifically designed to address IPV for nurses.

In their concept analysis of care, Cutcliffe and McKenna [5] defined four critical elements of caring: serious attention, concern, and providing for and getting to know the patients. Having respect for the patients must be present in order for caring to take place.

In the case of encountering women exposed to IPV, sufficient preparedness requires both knowledge and experience to identify victims of IPV and implement the right nursing interventions. Such interventions may include providing the correct information about the resources available and follow ups in the form of routine appointments or telephone calls. Walton-Moss and Campbell [28] also pointed out the importance of coordinating different interventions since women exposed to IPV often become isolated as a result of the abusers' controlling behaviour. For that, an integrated approach to health, welfare and justice system preparedness is needed.

Since IPV is common and has a profound impact on women and children's health primary health care professionals must become competent in identifying victims of IPV and offer them appropriate interventions. IPV in the context of PHC has only recently been better explored. Studies have explored the effect of education in the context of PHC but studies including district nurses in primary health care centres (PHCC) are missing [17,28-32]. Hence, understanding the nurses' educational needs would be valuable for designing educational programmes for nurses working in PHC. Increased preparedness in this group because nurses in PHC are keys to the identification of women exposed to IPV as their work with families and women of all ages often for long periods at a time, would present them with ample opportunities to detect IPV and intervene. Taking all these into account, the aim of the study was to assess nurses' preparedness to identify and provide nursing care to women exposed to IPV who attend primary health care.

## Methods

### Design

A questionnaire that measured nurses' preparedness in encountering with women exposed to IPV, (i.e. identifying them and provide nursing interventions) was developed, based on a systematic literature review [33] and the authors' knowledge and experience in this area. A draft was sent to a professional survey designer at Statistics Sweden who made modifications to it. The amended version consisting of 27 questions (including demographic) was pilot-tested by six nurses working in PHC who were asked to complete the questionnaire and comment on clarity and relevance of each question. Upon evaluating the returned questionnaires, two questions were removed; 'Do you participate in any kind of collaboration related to IPV?' and 'Do you have suggestions on training you think are important to your work with women facing IPV?' and two new questions were added; 'Are you a nurse or a district nurse?' and 'How many years have you worked as a nurse?'. The improved version of the questionnaire, still consisting of 27 questions (including demographic), was further tested on 39 nurses working in PHC in another county who were asked to comment on content, clarity and relevance. These nurses were not included in this study.

Final evaluation produced the final version of the questionnaire which consisted of 29 questions, nine of which aimed at assembling demographic data and personal experiences of IPV. The remaining 20 questions aimed at assessing the nurses' knowledge on IPV. No new questions were added after the second pilot-test, but two questions were divided.

1. The nurses' demographic data and experiences (9 questions): sex, age, birth country, profession, numbers of years working as a nurse, years as a district nurse, years at the current workplace, any personal experience of IPV.

2. The nurses' preparedness to provide nursing care to women exposed to IPV (20 questions):

- conditions at the organisation (7 questions): the nurses' working conditions, working environment and guidelines, special responsibility and interest, cooperation with other professionals and organisations and attitudes towards cooperation.

- personal attitudes (13 questions): the nurses' attitudes and knowledge on IPV, self-rated sufficient preparedness, knowledge and education, ability to identify IPV, attitudes towards asking (including reasons for not asking, if applicable) and frequency of asking (several alternatives could be ticked), preferred intervention implemented when suspicion of IPV was confirmed and also when it was not confirmed (several alternatives could be ticked).

Examples of answering alternatives were: 'yes', 'no' or 'do not know', and 'agree perfectly', 'agree somewhat', 'agree to some degree' and 'do not agree at all'. There was designated space at the end for nurses' to give comments when desirable. Analysis of estimated sample size were performed, using the key question 'If you suspected that a woman was exposed to IPV, would you confirm it by asking her if it was true?', and yielded a power of 90%. In total 125 participants were needed. Estimation of sample size was done to reach a 21% difference between those who could identify women exposed to IPV and those who could not.

### Setting and data collection

At the time of the study there were 174 PHCC across urban and rural areas in Stockholm County that employed nearly 1,200 active nurses. Of the 174 PHCC, 40 were randomly selected. During the randomisation process, every PHCC was given a unique number that was written on a paper card and placed in a pot. For transparency, two colleagues independently drew 20 paper cards each, a total of 40. All PHCCs selected were then contacted and were invited to participate in the study. One of the 40 PHCCs declined to participate. The nurses in each PHCC were contacted by telephone and the nurse who replied was asked to act as a contact person for the study. The nurses received verbal and written information about the study. They were asked to distribute the questionnaires and an information letter to their colleagues at their workplace, and to collect the sealed envelopes with the questionnaires after completion and send them to an independent person at the research centres. The questionnaires were coded so that they could be easily traced and reminders were sent to invite participants to follow-up interviews.

### Data analysis

The data were analysed using statistical software STATA 9.0. Descriptive statistics in the form of frequency tables were generated to describe the data in terms of number and percentage distribution. The summary and frequency tables were used to examine all variables used in the study. Pearson's chi-square test was used to test the statistical significance of the findings. A *p*-value of < 0.05 was indicative of statistical significance. Owing to the low number of answers to some answer alternatives, the options *always*, *sometimes *and *never *were dichotomised into two groups, *always *and *sometimes/never*.

Are the nurses prepared to identify and provide nursing care to women exposed to IPV who attend primary health care? To address this question, a two-step multivariate logistic regression analysis was performed. In step 1, 'if you suspected that a women was exposed to IPV, would you confirm it by asking her if it was true?' was used as the dependent variable, to assess nurses' ability to identify women exposed to IPV. In step 2, 'do you believe that you are sufficiently prepared to deal with a woman exposed to IPV?' was used as the dependent variable to find predictive factors associated with nurses' preparedness to deal with women exposed to IPV.

### Ethical considerations

Ethical approval was obtained from the Regional Ethical Reviews Board at Karolinska Institutet, Stockholm. All participants were fully informed that their data would not be passed on to any third parties, their participation was voluntary and anonymous and that they could withdraw any time. Furthermore, participants were forewarned that the study could stir up distressing memories of abuse (applicable to those with personal experiences with IPV). A list of centres to seek psychological support was also provided.

## Results

**Figure 1:**
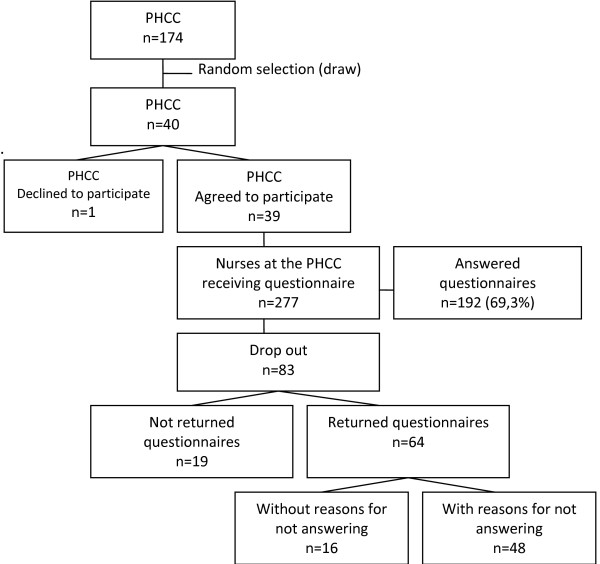
**Participant recruitment flowchart**.

Questionnaires were distributed to 277 nurses working at the 39 PHCC. The response rate was 69.3% (n = 192) after one reminder. Eighty-three nurses dropped out, 19 of whom did not return the questionnaire whilst 64 did. Of those 64, 48 provided reasons for not wishing to participate (i.e. lack of time, illness, holiday or maternity leave) whilst 16 returned the questionnaire unanswered (see Figure 1). Generally, the internal dropout was between 0% and 5% except for questions 'do you ask women if they are exposed to IPV when you suspect it?' and the question about 'Nurses' views on common attitudes toward Intimate Partner Violence' which showed an internal dropout of 9%.

Demographic data, working experience and personal experience with IPV based on completed questionnaires by the respondents are shown in Table 1. All respondents (n = 190), except one were women. Most of the respondents were born in Sweden (87%, n = 167), the mean age was 49 and mean number of years in the nursing profession was 21.

**Table 1:** Nurses demographic data and personal experiences of Intimate Partner Violence (IPV)

Demographic variables	n	(%)	sd	mean
**Sex (n = 190)**			0.725	
Female	189	(99)		
Male	1	(1)		
**Age (n = 183)**			11.219	49
20-39	24	(13)		
40-59	128	(70)		
≥ 60	31	(17)		
**Birth country (n = 186)**			0.651	
Sweden	162	(87)		
Nordic countries (Sweden excluded)	11	(6)		
Outside the Nordic countries	13	(7)		
**Numbers of years working as a nurse (n = 189)**			10.369	21
0-9	37	(19)		
10-29	107	(57)		
≥ 30	45	(24)		
**Numbers of years working as a district nurse (n = 138)**			6.442	12
0-9	62	(45)		
10-29	72	(52)		
≥ 30	4	(3)		
**Numbers of years at current working place (n = 188)**			5.494	6
< 1	19	(10)		
1-9	131	(70)		
10-19	25	(13)		
≥ 20	13	(7)		
**Nurses with personal experience of IPV (n = 191)**	23	(12)	0.336	
**Nurses with relative or near relation with experience of IPV (n = 191)**	58	(30)	0.664	

### Preparedness to provide nursing care for women exposed to IPV

#### Conditions at the organisation

At the 39 PHCC, 28 nurses (15%) had had previous discussions about how to intervene when meeting with women exposed to IPV, 10 (5%) had used existing guidelines and 47 (25%) had had information packages at their disposal to hand out to women exposed to IPV. Thirteen (7%) nurses stated that there was a specially assigned person at their workplace responsible for quality improvement of nursing care, one of whom was a district nurse.

A total of 132 (70%) nurses stated that they were not aware of any collaboration with the authorities in dealing with IPV and 165 (92%) considered such collaboration necessary. Fifty-eight (30%) acknowledged the presence of a collaboration, and named volunteer organisations and psychiatric and social services to be their collaborators.

Participants were asked whether their personal attitudes aligned with the general societal views on IPV (see Table 2). The predominant views on the nature of the perpetrator among the participants were that 'alcohol and drugs are common reasons that men abuse' and 'the perpetrator simply loses control'.

**Table 2:** Questionnaire

Part 1. Nurses' views on common attitudes toward Intimate Partner Violence (IPV).				
Attitudes	*Agree to some degree%	**Does not agree at all%	No opinion%	
Alcohol and drugs are common reasons for IPV (n = 182)	91	8	1	
The perpetrator simply loses control (n = 180)	69	25	16	
IPV is most common among the lower socioeconomic groups (n = 178)	25	71	4	
Victims of IPV can always leave the perpetrator if they want to (n = 181)	22	77	1	
For children's sake, it is important to keep the family together even when IPV occurs (n = 180)	12	86	2	
It is the victim's fault that she has been abused (n = 182)	3	97	1	

**Part 2.**				

Own preparedness			n	(%)

Have you obtained knowledge about IPV on your own?				
Yes			86	(48)
No			95	(52)
Total			181	(100)
Did you receive training about dealing with IPV in your vocational training?				
Yes			37	(20)
No			146	(80)
Total			183	(100)
In your professional work over the last three years, have you received any training on IPV?				
Yes			15	(8)
No			173	(92)
Total			188	(100)
Do you believe that you are sufficiently prepared to deal with a women exposed to IPV?				
Yes			26	(14)
No			158	(86)
Total			184	(100)
Are you interested in learning about IPV and how to deal with it?				
Yes			147	(82)
No			33	(18)
Total			180	(100)
In which country did you receive your nursing degree?				
Sweden			184	(3)
Nordic countries (Sweden excluded)			5	(3)
Outside Nordic countries			1	(0)
Total			190	(100)

**Part 3. **Nurses' description of signs that indicate IPV. Several alternatives were available				

Signs that indicate IPV			n	(%)

The woman's explanation is not consistent with the injury			145	(76)
Bruises			134	(70)
The partner is overprotective or refuses to leave the woman alone with the nurse			129	(68)
Injuries to the face, arms and/or torso			128	(67)
Hair pulled out			118	(62)
Earlier A&E visits with injuries of an unclear nature			114	(60)
The woman waited a long time to seek help for the injuries			104	(54)
The woman comes frequently for diffuse complaints with no improvement			100	(52)
Bilateral or multiple injuries on the same or different dates			94	(49)
An injured pregnant women			91	(47)
Mental/psychosomatic problems			90	(48)
Fractures			76	(40)
Sleeping disorders			75	(39)
Injuries to the lower part of the body			73	(38)
Burns			68	(36)
Difficulties coping with a physical examination			64	(34)
Puncture wounds			63	(33)
Chronic pain without distinct reason			58	(30)
Gastrointestinal disorders			46	(24)
High or low BMI			40	(21)

**Part 4. **The interventions the nurses stated that they carried out when they *suspected *or when they *knew *that a woman was exposed to IPV. Several alternatives were available.				

List of interventions			Suspected n (%)	Knew n (%)

Offer her an appointment with a doctor			127 (68)	137 (74)
Meet the women alone, without her partner			111 (60)	104 (56)
Give her information about volunteer organisations, such as women's shelters, crime victims hotline			104 (56)	134 (72)
Notes in the patient records such as nurses' observations and suspicions			100 (54)	13 (61)
Ask her if she has children			98 (53)	107 (58)
Ask her about her relationship with the man I suspect abuses her			81 (44)	97 (52)
Try to find out what kind of abuse she was exposed to (physical, mental, economic/financial abuse etc.)			80 (43)	103 (55)
Use an authorised interpreter if the women cannot speak Swedish language			79 (42)	114 (61)
Ask about her social background (relationships, social networks, profession etc)			79 (42)	96 (52)
Offer her a follow-up appointment			72 (39)	70 (38)
Offer her an appointment with someone else at the health centre for follow-up talks			50 (24)	51 (27)
If the women has children under age, report to social services that a child may be at risk			44 (24)	71 (38)
Listen to her description of the violence she was subjected to				131 (70)
Advise her to contact the police				112 (60)
Offer her help contacting the social services				84 (42)
Offer her help contacting the police				79 (42)
Offer to call her later				53 (28)
Offer her a home visit				27 (15)

* These answers include the following alternatives: 'agree perfectly', 'agree somewhat' and 'agree to some degree'

** The preferred answer on all questions

When participants were asked whether they had received any kind of training, 86 (48%) stated that they had obtained information on their own initiative, 37 (20%) had had vocational training and 15 (8%) had received training on their employers' initiative. The majority of those who obtained information on their own initiative had actively searched the information in the mass media and literature due to personal commitment and interest in the area. A total of 158 (86%) considered themselves to be insufficiently prepared to provide nursing care to women exposed to IPV and 147 (82%) were interested in receiving training to increase their competence in this area (see Table 2). In total 26 (13%) offered comments on putative reasons for insufficient preparedness, such as lack of experience, training, continuing education opportunities, resources, guidelines and cooperation with other authorities in the community. A total of 191 nurses answered the question about whether they had themselves experienced IPV, 23 of whom (13%) reported that they had. Additionally, 58 (30%) of the 191 nurses had either a relative or near relation who had been exposed to IPV.

In answering questions on frequency of their encounters with women exposed to IPV, 18 (10%) stated that they encountered victims of IPV once a week to once a month, 137 (73%) less than once a month and 32 (17%) had never encountered IPV victims. Participants understood that IPV was evident when the women's account of events was not consistent with their injuries, when the women had bruises and when the women's partners appeared over-protective. Chronic pain without an obvious cause, gastrointestinal symptoms and high or low BMI (see Table 2) were considered to be lowest manifestations of the abuse.

Regarding their personal attitudes towards asking direct questions about suspected IPV, 174 of the 192 participants (91%) completed the respective questions. Of these, 90 (52%) stated that they always asked and 84 (48%) sometimes/never. Fifty-seven (32%) of the 84 who sometimes/never asked stated that it was difficult to know how to ask the question, 19 (11%) that they did not know what to do with the answer, 23 (13%) that they were worried about breaching the woman's integrity, 18 (10%) that they did not have time, 8 (4%) that they felt uncomfortable about touching this issue, and 5 (3%) that they did not want to get involved in a private matter. Participants had ticked several alternatives as they were allowed to do so in this case.

The preferred interventions of the participants when they *suspected *IPV or *knew *the woman had been exposed to IPV are shown in Table 2. The three most common interventions on *suspicion *were doctor referrals ensuring that women had privacy and providing information on volunteer organisations. On average, five out of 12 possible interventions were used by all participants when they suspected IPV. The three most common interventions on *knowing *were doctor referral, providing information on volunteer organisations and listening to the woman's story. The least common preferred interventions were reporting the situation to the social services (when children were exposed), offering follow-up visits at the PHCC, and home visits. On average, nine out of the 18 possible interventions were used by the participants when they knew IPV had occurred.

### Factors associated with the identification of women exposed to IPV

'Being sufficiently prepared' was found to be the only significant independent variable in the first step of multivariate logistic regression analysis (p = 0.002). Nurses were six times more likely to ask about IPV if they felt sufficiently prepared. Several models were tested and only the age-adjusted model was statistically significant. Age adjustments resulted in grouping the participants in three age categories. The eldest were > 60 years old. The age-adjusted odds ratio (OR) was 6.30 (95% CI 2.02-19.67) and referred to whether nurses identified women exposed to IPV and whether they felt sufficiently prepared (see Table 3).

**Table 3:** Multivariate logistic regression with factors associated with nurses' identification of women exposed to Intimate Partner Violence (IPV), i.e. stating that they asked women about violence

'If you suspected that a woman was exposed to IPV, would you confirm it by asking her if it was true?'	Odds Ratio	P > |z|	[95% Conf. interval]
Not sufficiently prepared to deal with a women exposed to IPV	1 (ref)		
Sufficiently prepared to deal with a women exposed to IPV	6.30	0.002	2.02-19.67
Age 20-39	1.64	0.315	0.62-4.31
Age 40-60	0.89	0.854	0.27-2.93
Age > 60	1 (ref)		

In the second step, being sufficiently prepared was used as a dependent variable to find predictive factors (see Table 4). Only 'having obtained knowledge by themselves' was a significant independent variable (p < 0.001). Variables 'being sufficiently prepared' and 'having obtained knowledge by themselves' were shown to be closely associated reflected by an OR of 7.53 (95% CI 2.46-29.03). Nurses were nine times more likely to ask about violence if they had obtained information about violence by own initiative. The age-adjusted odds ratio for the association was 9.07 (95% CI 2.83-29.13).

**Table 4:** Multivariate logistic regression with factors associated with nurses' preparedness to meet women exposed to Intimate Partner Violence (IPV)

Yes on the question: 'Do you believe that you are sufficiently prepared to deal with a woman exposed to IPV?'	Odds Ratio	P > |z|	[95% Conf. interval]
'Did you receive training about dealing with IPV in your vocational training?' and/or 'Did you receive training about dealing with IPV in your professional work?'	1 (ref)		
'Have you obtained knowledge about IPV by own initiative?'	9.07	0.01	2.82-29.12
Age 20-39	0.38	0.26	0.09-1.50
Age 40-60	0.57	0.46	0.11-2.84
Age > 60	1 (ref)		

## Discussion

### Summary of the main findings

In summary, the results implied shortcomings regarding preparedness to care for women exposed to IPV among the nurses included in the study. Specifically, shortcomings were found both at the level of the organisation and the individual. Many had poor knowledge of the issues around IPV and shared attitudes and views similar to those of other people in their community. Only half of them stated they always asked women about IPV when IPV was suspected but only did so when woman showed visible injuries. Participants were generally unsure of how to ask direct questions, and when they identified a women exposed to IPV, they referred them to a physician as their preferred intervention method. The results also indicate that feeling prepared meant having obtained knowledge which influenced the nurses' ability to identify women exposed to IPV. These shortcomings may lead to delays in offering the appropriate care for women exposed to IPV.

### Preparedness to provide nursing care to women exposed to IPV

Several shortcomings were found regarding support at the level of the organisation. Our results highlighted lack of relevant mandate to deal with the issue which was reflected in a lack of guidelines on proper nursing care and cooperation with other authorities. Several studies have shown that organisational support is important to improve care of women exposed to abuse. According to Minsky-Kelly et al. [34], support in the form of continuing education along with guidelines are necessary steps to be taken by a health provider to improve care of women exposed to IPV. Waalen et al. [35] showed that education increased preparedness in identifying women exposed to IPV and that strategic interventions coupled with training improved screening rates. In addition, another study showed that guidelines had an impact on the nurses' willingness and ability to ask women about violence and on to properly manage their care [36].

Although guidelines are meant to facilitate IPV detection and implementation of the appropriate intervention methods, results from several studies are so far inconclusive [17,32,33,35]. At the same time, it is well known that only when guidelines are implemented in the organisation can nurses effectively support women exposed to IPV [37]. When guidelines are, therefore, lacking nurses may have to improvise with uncertain outcomes [38]. In this study, only 5% of the participants stated they were aware of written guidelines. It was not known whether this was due to complete lack of guidelines or lack of knowledge about existing guidelines. Either way, special attention should be paid on the impact lack of guidelines or lack of awareness of existing guidelines has on the nurses' self-rated preparedness. In this study, nurses considered the lack of guidelines as inhibiting in dealing with women exposed to IPV.

Conditions at the organisation and personal attitudes towards IPV equally affect nurses' preparedness. When nurses were asked to explain the reasons for not asking women about violence many stated that they felt uncomfortable about addressing the issue. Awareness of one's own attitudes plays an important role in one's preparedness to deal with abused women [16]. Participants' attitudes towards women exposed to IPV matched general societal views and was consistent with findings by others [37]. Training programmes must deal with this problem, since having the 'prejudicial' attitudes is known to negatively impact the nurses' interaction with abused women as well as their ability to identify them and properly care for them [37]. As an example, almost every fourth respondent in this study agreed with the general notion that "*Victims of IPV can always leave the perpetrator if they want to*". This view could have a significant impact on the nurses' decision of whether to ask women about violence or not. One study reported that in-service training gave the nurses a better understanding of the difficulties women have in leaving their abusive partners. Knowing the difficulties for women to leave the perpetrator may have a positive impact on the nurses' encounters with women exposed to IPV and the quality nursing care they could offer them. Many nurses in the present study stated a need for training which was found to be closely associated with 'feeling sufficiently prepared' to identify abused women.

In previous studies, women exposed to IPV preferred to be asked directly about IPV [39-41]. In this study, approximately half of the nurses stated that they always did so when they suspected IPV. This rate seems to be higher than that reported in earlier studies, except that of nurses working in obstetrics and gynaecology [42]. Overall, studies have shown that very few women are asked directly about IPV by health care professionals [25,43-46]. This might also be the case for the population in this study, since, even though half of the respondents stated that they always asked women about IPV when they suspected it, most of them met women exposed to IPV less than once a month. This could result in lack of knowledge about health problems caused by IPV, which, in turn, may lead to cases of IPV remaining undetected. This is supported by the finding that nurses suspected IPV only when women had visible injuries, which is a common misunderstanding about health problems caused by IPV [47,48]. The most common health effects are psychological and psychosomatic problems such as depression and chronic pain without an obvious cause. In the present study, these signs did not cause suspicion as often as bruises and injuries did. Furthermore, this is probably closely associated with lack of knowledge. Interestingly, few of the nurses in this study had received training about IPV during their professional education or during their employment. It is, thus, not surprising that those who could identify IPV victims are those who had obtained knowledge on their own initiative.

This study showed that 13% of the nurses had personal experiences with IPV and one out of three had a relative or near relation who had been exposed to IPV. Even though there was no indication of an association between having experienced IPV and higher odds of successfully identifying IPV victims, the personal experience may influence the quality of nursing care given to IPV victims [48].

The word 'caring' in nursing care, means 'providing for', and there are many nursing interventions that should be considered when caring for women exposed to IPV [5]. The questionnaire suggested 18 possible interventions the nurses could choose from when encountering a woman they knew had been exposed to IPV. However, only two nurses reported that they had used all 18 interventions. Nurses' most common intervention was to refer the woman to a doctor. A doctor's appointment is a necessary intervention but it might also, be a way of 'passing the buck' when one is not aware of other nursing interventions, does not have written guidelines, and/or feels uncomfortable encountering someone who has been exposed to IPV. It could also mean that nurses believed that doctors were more prepared to intervene in cases of IPV which may also be the case sometimes but not always.

Caring for an abused woman may also include caring for her children who may also been exposed to violence. It is commonly known that IPV affects children's health just as much as it affects the women's. Nurses are obligated to report children living in an abusive home to social services. However, very few nurses stated that they reported these families to social services. This is a potentially serious shortcoming, as the families where IPV occurs need considerable support. Nurses also need to respond appropriately to women's disclosure, as it seems that the effect of distressful disclosure experiences may lead to a gradual reduction in health care seeking among women exposed to IPV [49].

### Study limitations

Analysis of estimated sample size was performed and yielded a power of 90%. Totally 125 participants were needed and in fact, 192 nurses participated, why the power was judged sufficient. The confidence intervals in the regression models were rather wide, which could indicate a low statistical power. However, the response rate was 69.3% which must be considered large in this kind of a study, and the calculated power to detect a significant difference in the study sample was 90%, which is satisfactory. Among the 83 nurses who did not answer the questionnaire reasons for this are known for more than half of them since they returned the questionnaire with written comments regarding this. However, the nurses, in total 35, who did not return their questionnaire or returned it but gave no written reasons for not answering, were not further contacted. It is therefore not known if they introduce a selected bias. This was, however, a decision taken from an ethical point of view and out of respect for the nurses' privacy, since IPV is considered sensitive. The internal dropout was between 0% and 5% except for two questions where it was 9%, even though it must be considered as low. The decision was made to not exclude the questionnaires or the variables with missing values since they occurred randomly and did not affect the outcome of the study.

## Conclusions

The majority of the nurses were found to be quite unprepared to provide nursing care to women exposed to IPV, and the majority of them felt unprepared as well. This is problematic, particularly because feeling prepared was found to be associated with the ability to identify women exposed to IPV. Reduced preparedness was also associated with lack of knowledge and only those nurses who had sought knowledge on their own initiative appeared assertive in dealing with the women exposed to IPV. There was little organisational support - the majority lacked written guidelines and collaboration, as well as basic knowledge about signs of IPV, how to identify victims and how to intervene. These shortcomings can lead to inappropriate care for the women exposed to IPV and their children. Improvements are needed at both organisational and individual levels. These findings could be used to develop educational programmes for nurses working in PHC so that they will be better prepared to care for women exposed to IPV.

## Competing interests

The authors declare that they have no competing interests.

## Authors' contributions

ES, NSS and LT were responsible for the study conception and the design and drafting of the manuscript. ES performed the data collection and the data analysis. PW were helpful in the discussion of statistical methods and the data analysis as well as drafting the manuscript. All authors read and approved the final manuscript.

## Pre-publication history

The pre-publication history for this paper can be accessed here:

http://www.biomedcentral.com/1472-6955/11/1/prepub
